# Gene Expression and Alternative Splicing Regulate Phenotypic Plasticity of a Social Wasp

**DOI:** 10.1002/ece3.73930

**Published:** 2026-07-13

**Authors:** Michael A. Catto, Sarah E. Orr, Brendan G. Hunt, Michael A. D. Goodisman

**Affiliations:** ^1^ School of Biological Sciences Georgia Institute of Technology Atlanta Georgia USA; ^2^ Department of Entomology University of Georgia Griffin Georgia USA; ^3^ Department of Biology University of Tampa Tampa Florida USA

**Keywords:** caste‐specific profiles, genetic variation, heritability, social insects, tissue‐specific profiles, *Vespula maculifrons*

## Abstract

Phenotypic plasticity enables a single genome to produce multiple highly variable phenotypes. Plasticity arises through transcriptomic variation, which includes differential gene expression and alternative splicing. Here, we use the polyandrous social wasp *Vespula maculifrons* to examine genetic and environmental effects on transcriptome variation and phenotypic plasticity. To assess transcriptomic diversity, we quantified differential gene expression and alternative splicing between head and thoracic tissues of queens and workers. Gene expression differences were stronger across tissues than castes and exceeded differences observed for alternative splicing. We uncovered significant correlation between differential gene expression and alternative splicing patterns, indicating these two regulatory layers are partly overlapping. We also found that genetic background within a colony influenced transcriptomic variation, but to a much lesser extent than colony membership. This result is consistent with stronger effects of environmental variation than genetic variation in shaping transcriptomic variation. Together, our results demonstrate how multiple layers of transcriptome regulation vary in the context of developmental differentiation, colony environment, and genetic lineage of a natural social system.

## Graphical abstract

This study demonstrates that patterns of differential gene expression and alternative splicing are partially linked in a social wasp. In addition, colony‐level environmental variation drives far stronger transcriptomic divergence than genetic differences, highlighting that social environment plays a strong role in shaping developmental plasticity.

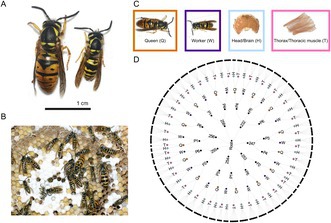

## 1 Introduction

Phenotypic plasticity arises from environmentally responsive regulation of gene activity. This regulatory variation manifests as changes in the transcriptome, including changes in gene expression levels and shifts in alternative splicing (Jones et al. 2024; Marasco and Kornblihtt 2023). Understanding how transcriptomic variation differs across developmentally produced phenotypes is essential for elucidating the molecular mechanisms that enable organisms to produce phenotypes suited for different environments.

Despite the recognized importance of transcriptomic variation in generating phenotypic diversity, fundamental questions about the architecture of phenotypic plasticity remain unresolved. For example, the relationship between differential gene expression and alternative splicing as regulatory mechanisms remains unclear (Verta and Jacobs 2022). If a gene is both differentially expressed and alternatively spliced, changes in abundance and isoform composition may jointly influence phenotype. Conversely, if differentially expressed genes rarely overlap with alternatively spliced genes, then splicing may regulate phenotypes through mechanisms decoupled from transcript abundance. Clarifying the relationship between these regulatory processes is necessary, and exploring how gene expression and alternative splicing interact is essential for understanding how regulatory mechanisms shape phenotypic plasticity (Jones et al. 2024; Luo et al. 2025; Marasco and Kornblihtt 2023; Singh and Ahi 2022; Verta and Jacobs 2022).

Additionally, the relative contributions of genetic and environmental variation to transcriptomic differences are not well characterized, particularly in natural populations. Environmental variation can lead to substantial variation in patterns of gene expression and alternative splicing, and is a fundamental component to the generation of alternate phenotypes through phenotypic plasticity (Gracey 2007; Hodgins‐Davis and Townsend 2009; Marais et al. 2013; Rivera et al. 2021). At the same time, genetic effects arising from DNA sequence variation can influence both gene expression and alternative splicing (Aguet et al. 2017; Castaldi et al. 2022; Garrido‐Martín et al. 2021; Grantham and Brisson 2018; Kim and Wysocka 2023; Minow et al. 2023; Monlong et al. 2014; Wong et al. 2025; Yamaguchi et al. 2022). However, studies that partition transcriptomic variation into genetic and environmental components in natural populations remain rare, limiting our understanding of how these factors interact to shape phenotypic plasticity.

Social insects provide powerful models for studying how regulatory variation in the form of differential gene expression and alternative splicing generates phenotypic diversity. Insect societies consist of discrete castes that show strikingly distinct phenotypes despite sharing a common genome (Wilson 1971; Wilson and Hölldobler 2005). In hymenopteran social insects, which include ants, social bees, and social wasps, the queen and male castes mate and reproduce while the worker caste undertakes activities related to foraging, nest building, nursing, and colony function (Oster and Wilson 1978; Wilson and Hölldobler 2005). Caste differentiation in social insects thus provides an excellent system for studying how transcriptomic regulation generates discrete phenotypes from shared genomes (Pyenson and Rehan 2024; Robinson et al. 2005; Taylor et al. 2019; Treanore et al. 2021; West‐Eberhard 2003; Wilson 1971; Kocher and Kingwell 2024; Okwaro and Korb 2023). Many studies have investigated how differences in gene expression are associated with caste differences (Berens et al. 2015; Feldmeyer et al. 2014; Kocher and Kingwell 2024; Luo and Zhou 2025; Oldroyd and Yagound 2021; Orr and Goodisman 2023; Pyenson and Rehan 2024; Sumner et al. 2018). More recently, studies have begun to examine how post‐transcriptional splicing variation is associated with phenotypic variation between insect castes (Awde et al. 2022; Ding et al. 2025; Gao et al. 2020; Harrison et al. 2022; Holman et al. 2019; Luo and Zhou 2025; Marshall et al. 2019; Miyazaki et al. 2021; Price et al. 2018; Zhu et al. 2021).

The purpose of this study is to understand the sources of variation that shape transcriptomic variation. We specifically address questions related to the interaction of gene expression and alternative splicing using a highly social wasp as a model system. First, we explore how caste and tissue identity shape gene expression patterns. We assess whether caste‐associated expression differences are pervasive across the organism or instead concentrated in specific tissues relevant to reproductive and behavioral specialization. Second, we study patterns of alternative splicing in the context of developmental plasticity, testing whether isoform variation is structured by caste and tissue identity in ways that parallel or diverge from expression‐level regulation. Alternative splicing has been insufficiently characterized as a regulatory process in natural populations. Third, we examine the relationship between differential expression and alternative splicing to determine whether these mechanisms act independently or are coordinated within the same genes. Specifically, we ask whether differential gene expression and alternative splicing could be viewed as complementary mechanisms of regulation contributing to phenotypic plasticity at multiple regulatory levels. Finally, we assess the effects of genetic and environmental variation on gene expression and splicing diversity, asking how much regulatory variation arises from colony‐level genetic differences versus environmentally induced plastic responses. Few studies have partitioned genetic and environmental effects on transcriptomic variation in natural populations, particularly in systems with extreme developmental plasticity. By integrating these hierarchical levels of analysis, our research provides insight into the multiscale factors that shape transcriptomic variation within species, from gene regulation within tissues to genetic and environmental variation among individuals and colonies.

## 2 Methods

### 2.1 Target Species and Sampling Strategy

**FIGURE 1:**
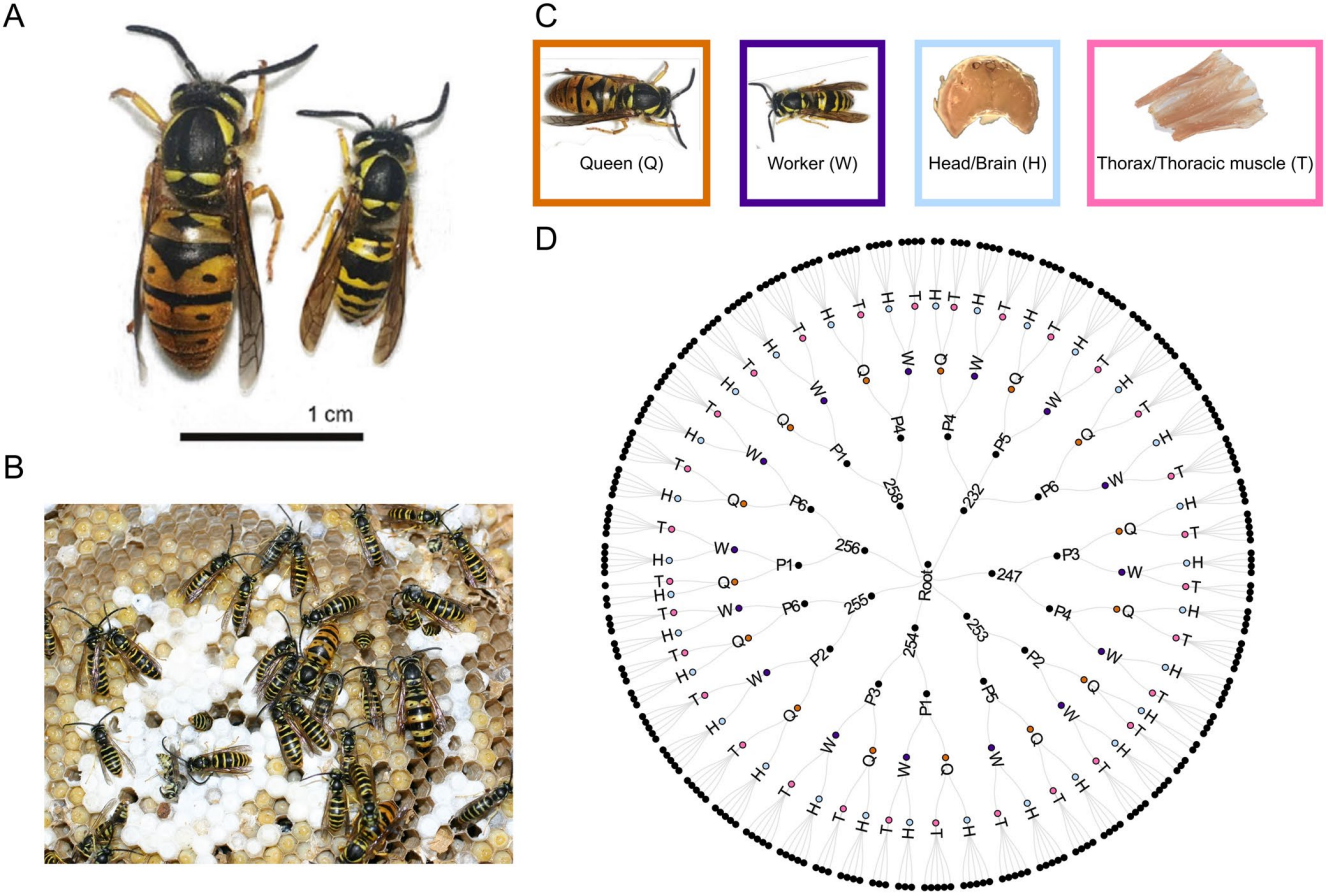
The social wasp *Vespula maculifrons* and overview of study design. (A) Queen and worker *V. maculifrons* wasps display substantial caste differences. (B) *V. maculifrons* nest showing different castes. (C) *V. maculifrons* castes and tissues used for transcriptomic analyses in this study. Tissue images are not to scale. (D) Hierarchical sample wheel showing the sample attributes. From the center to the outer layer, the order of attributes is colony (*n* = 7), patriline (*n* = 2–3 per colony), caste (*n* = 2 per patriline), tissue (*n* = 2 per caste), and sample (*n* = 1–6 per tissue; terminal node).

Our study system is the Eastern Yellowjacket wasp, *Vespula maculifrons* , a highly abundant social wasp native to eastern North America (Hunt 2007; Lester and Beggs 2019; MacDonald and Matthews 1981; Ross and Matthews 1991; Sumner 2022). *V. maculifrons* queen and worker castes differ substantially in morphology, physiology, and behavior (Figure 1A). Individual *V. maculifrons* colonies are founded by a single queen who produces all of the colony's offspring (Goodisman et al. 2007a, 2007b; Kovacs et al. 2010; MacDonald and Matthews 1981) (Figure 1B). Individuals from different colonies therefore differ genetically and experience distinct environments (Khaliq et al. 2014). Within colonies, workers and new queens also differ genetically because *V. maculifrons* queens mate with multiple males (Goodisman et al. 2007b; Orr, Hedrick, Murray, Pasupuleti, and Goodisman 2024). Therefore, workers and new queens within colonies consist of either full siblings (members of the same patriline) or half‐siblings (members of different patrilines).

*V. maculifrons* colonies were collected in November 2021 and 2022 from sites in the greater Atlanta, Georgia area (Orr, Hedrick, Murray, Pasupuleti, and Goodisman 2024; Orr, Hedrick, Murray, Pasupuleti, Kovacs, and Goodisman 2024). Entire nests were excavated and transported to the laboratory in ventilated plastic containers. Colonies were chilled at 4°C to immobilize individuals, which were then sorted into labeled tubes. We note that the process of sample collection may have affected subsequent patterns of gene expression. We preserved between 18–28 workers and 14–40 pre‐reproductive queens (i.e., gynes, hereafter referred to as “queens”) from seven individual colonies in liquid nitrogen. Samples were stored at −80°C until needed for molecular analysis.

### 2.2 Microsatellite Genotyping and Patriline Identification

DNA was extracted from leg tissue of queens and workers. Legs were flash frozen in liquid nitrogen, pulverized with a sterile pestle, and combined with 250 μL of 5% Chelex solution (Walsh et al. 1991). DNA solutions were incubated at 95°C for 30 min. DNA was then stored at −20°C until further use.

Individual queens and workers were genotyped at four polymorphic microsatellite loci using standard PCR techniques (Daly et al. 2002; Foster and Ratnieks 2001; Hasegawa and Takahashi 2002) (Table S1). PCR products were visualized on 3% agarose gels with ethidium bromide to confirm amplification. Then, samples were combined with a ROX‐labeled size standard and formamide, denatured at 96°C for 3 min, and snap cooled on ice. Fragment analysis was conducted by Eton Biosciences. Microsatellite allele scoring (Table S2) was performed manually using ThermoFisher Connect Microsatellite Analysis software. We found that microsatellite markers were highly polymorphic; patriline non‐detection error was estimated to be 0.000249.

We genotyped 275 individuals in total (147 queens, 128 workers) from each of the seven colonies. We identified 5–8 patrilines per colony (Orr, Hedrick, Murray, Pasupuleti, and Goodisman 2024). For downstream analyses, we retained individual queens and workers from the 2–3 most frequent patrilines per colony, ensuring a minimum of two individuals per caste–patriline combination. Our sampling design allowed us to quantify transcriptomic variation among colonies, patrilines, castes, and tissues (Figure 1C,D).

### 2.3 RNA Extraction, Library Preparation, and Sequencing

We investigated patterns of gene expression among castes and tissues of *V. maculifrons* . In total, 127 genotyped individuals were dissected into head and thoracic tissue using sterile blades. Legs and wings were removed from thoracic samples, while heads were kept intact with antennae attached. All dissections were stored at −80°C until RNA extraction.

RNA was isolated from each tissue using the Zymo Quick‐RNA Miniprep Kit, including the on‐column DNase treatment. RNA concentration and purity (260/280 ratio) were measured with a Nanodrop OneC. Samples were stored at −80°C before shipment on dry ice to the University of Georgia Genomics and Bioinformatics Core (GGBC) for RNA sequencing (Table S3). Our dataset included 253 RNA‐seq samples derived from 119 queens and 134 workers collected from seven colonies representing 15 distinct patrilines. Both head and thoracic tissues were sampled, yielding 127 head and 126 thorax transcriptomic libraries for tissue‐specific analyses.

RNA integrity was confirmed with a Qubit 4 Fluorometer. Poly(A)‐selected stranded RNA‐seq libraries were prepared and sequenced on an Illumina NextSeq2000. Sequencing occurred in seven batches: the first two used P1‐300 (*n* = 8) and P2‐200 (*n* = 11) flow cells, and the remaining five batches used P3‐200 flow cells (45–47 samples each). Each sample yielded ~3.75 Gb of data. Sequencing batch effects were accounted for in downstream analyses. Sample relationships were visualized using *data.tree* (Glur 2023), *shinyTree* (Allen 2023), *networkD3* (Gandrud et al. 2017), and *robustbase* (Todorov and Filzmoser 2009; Maechler et al. 2025).

### 2.4 Read Processing, Quality Control, and Mapping

Raw RNA‐Seq reads (~22M sequences per sample; Table S4) were trimmed with Trimmomatic v0.39 to remove adapters and low‐quality bases (sliding window = 4 bp, minimum quality = 15, minimum read length = 36 bp) (Bolger et al. 2014). TruSeq3‐PE adapters were clipped with the ILLUMINACLIP parameter (2:30:10), and bases with quality < 3 at read ends were removed (Table S5). Quality control was performed using FastQC v0.11.9 (Andrews 2010).

Reads were mapped to the *V. maculifrons* reference genome using STAR v2.7.10a (Catto et al. 2024; Dobin et al. 2013). Genome indexing was prepared with STAR and RSEM v1.3.3, with GFF annotations converted to GTF format via gffread v0.11.6 (Dobin et al. 2013; Li and Dewey 2011; Pertea and Pertea 2020). Mapping parameters allowed a maximum intron length of 1,000,000 bp, minimum intron length of 20 bp, and up to 20 multi‐mapping locations per read. Gene‐level expression was quantified with RSEM using paired‐end alignments, reverse strandedness, and read start position estimation (~63% mapping rate per sample; Table S6). MultiQC v1.14 summarized quality metrics (*n* = 253) such as raw read counts (*M* = 14.14M, SD = 10.25M), trimming efficiency (*M* = 12.97M, SD = 9.83M), and alignment statistics (*M* = 15.06 M, SD = 8.84 M) (Ewels et al. 2016). Transcripts per kilobase million (TPM), fragments per kilobase million (FPKM), and expected counts were computed for each sample; one low‐quality sample (253p2G13H) was removed.

### 2.5 Gene Expression Analyses

Raw counts were normalized with the variance‐stabilizing transformation in DESeq2 v1.30.0 (Love et al. 2014). Principal component analysis (PCA) of variance‐stabilized counts was performed with DESeq2 and PCATools v3.20 to assess variance structure (Blighe and Andrews 2019). One sample (247p3W3T) was excluded as it did not cluster well with the visually distinct cluster of worker thorax samples in PC1 vs. PC2 (Table S7). Correlations between principal components and biological traits were calculated to identify major drivers of transcriptomic variation.

Using DESeq2 normalized counts, we calculated the tau (*τ*) specificity index for each gene as *τ* = (∑(1 − (*x*_*i*_/*x*_max_)))/(*N* − 1), where *x*_*i*_ is the expression of a gene in sample *i*, *x*_max_ is the highest observed expression, and *N* is the total number of samples (Yanai et al. 2005) (Table S8). Tau ranges from 0, for genes that are expressed equally across all samples, to 1, for genes that are expressed solely in one focal sample.

DESeq2 was used to identify differentially expressed genes among castes, tissues, colonies, and sequencing batches. We fit multivariable generalized linear models with the design formulas ~Batch + Colony + Caste + Tissue (Table S9) and ~Batch + Patriline + Caste + Tissue (Table S10). These models simultaneously account for all specified factors, allowing us to test specific contrasts (e.g., queen vs. worker) while controlling for technical and biological covariates. Transcripts with a false discovery rate (FDR) < 0.05 after Benjamini‐Hochberg correction were considered significantly differentially expressed. Log2 fold changes were extracted for each contrast of interest, and genes showing significant differential expression were visualized using EnhancedVolcano v1.6.0 (Blighe et al. 2018). To summarize genome‐wide expression patterns, fold change distributions from major contrasts (queen vs. worker, head vs. thorax, and among colonies and patrilines) were binned and plotted using ggplot2 v3.3.5 (Wickham 2016).

To identify modules of co‐expressed genes, we applied weighted gene co‐expression network analysis (WGCNA v1.70.3) to variance‐stabilized expression values (Langfelder and Horvath 2008) (Table S11). Soft thresholding powers were selected to achieve scale‐free topology, ensuring that the resulting network exhibited biologically realistic properties. We constructed topological overlap matrices (TOM) to measure the similarity of gene expression patterns, then used hierarchical clustering to detect modules of highly co‐expressed genes. Module eigengenes (the first principal component of each module's expression profile) were correlated with biological traits to identify modules associated with caste, tissue type, or other experimental factors. To correlate principal components and module eigengenes with categorical traits, we encoded traits as binary numeric vectors (e.g., Caste: Queen = 0, Worker = 1) and calculated Spearman's rank correlation coefficients. Within significant modules, we identified hub genes based on high intramodular connectivity, which may represent key regulatory nodes. Network structures were visualized in Cytoscape v3.9.1 (Shannon et al. 2003).

### 2.6 Alternative Splicing Analyses

Alternative splicing patterns were analyzed using rMATS‐turbo (Wang et al. 2024), which detects differential usage of specific splice junctions and exons (Table S12). We employed additive models for gene expression (DESeq2) to account for multiple covariates simultaneously. For alternative splicing, we utilized rMATS‐turbo with pairwise comparisons. While a unified additive model for splicing would be ideal, current tools (including rMATS) do not support complex multi‐factor designs as flexibly as DESeq2. Thus, we prioritized statistical power for detecting subtle splicing shifts by aggregating replicates, acknowledging this as a trade‐off in experimental design. Alternative splicing analysis employed pairwise group comparisons. For each comparison, all samples from one group (e.g., all queens across colonies and batches) were compared against all samples from another group (e.g., all workers). This approach maximizes statistical power for detecting subtle shifts in isoform usage by aggregating biological replicates within each condition.

rMATS parameters were set to accommodate our experimental design: ‐‐readLength 100, ‐‐variable‐read‐length, ‐‐libType fr‐firststrand, ‐‐allow‐clipping, ‐‐novelSS, ‐‐cstat 0.05, and ‐‐t paired. These settings were optimized for 100‐bp paired‐end, strand‐specific libraries and enabled detection of both annotated and novel splice events. The analysis produced junction counts (JC) quantifying read support for different splicing outcomes (e.g., exon skipping, intron retention, alternative 5′ or 3′ splice sites), which were used to identify significant differences in splicing patterns between groups. We utilized the JC model, which calculates the Percent Spliced‐In (PSI) index values based solely on reads spanning splice junctions, providing a conservative estimate of inclusion levels less susceptible to mapping biases in exon bodies compared to the junction counts + exon counts (JCEC) model.

To compare the distribution of alternative splicing event types between experimental groups, we assembled counts of significant splicing events into contingency tables (rows = splice‐event type; columns = group comparisons of queen vs. worker or head vs. thorax). We performed Pearson chi‐square tests of independence in R v4.4.0 using chisq.test(…, correct = FALSE), reporting *χ* ^2^, degrees of freedom, and exact *p*‐values. The continuity correction was omitted because all expected cell counts exceeded five. We extracted standardized Pearson residuals from the test objects ($residuals) to identify event types contributing disproportionately to the overall association, where positive residuals indicate observed counts exceeding expected values and negative residuals indicate deficits.

### 2.7 Functional Enrichment

Gene‐set enrichment and semantic similarity analyses were performed on DESeq2, WGCNA, rMATS, and *τ* outputs using a custom R pipeline that integrates topGO (Alexa and Rahnenfuhrer 2026), rrvgo (Sayols 2023), and supporting Bioconductor resources. Gene identifiers were mapped to *Drosophila melanogaster* GO annotations via org.Dm.eg.db, and a universal genes‐to‐GO dictionary (gene2go.map) was loaded for downstream enrichment.

For each ontology (Biological Process, Molecular Function, Cellular Component), a binary gene list distinguishing significant versus background genes was constructed and fed into topGO. Classic Kolmogorov–Smirnov tests generate raw *p*‐values, which were subsequently corrected for multiple testing using both Benjamini–Hochberg FDR and q‐value estimations. Significant GO terms (FDR ≤ 0.05) were then passed to rrvgo, where pairwise semantic similarities were computed with the “Rel” method on the appropriate godata object. Redundant terms were collapsed using a similarity threshold of 0.7 and produced a reduced term matrix (Table S13).

### 2.8 Relationship Between Gene Expression and Alternative Splicing

We first tested whether genes exhibiting differential expression overlapped significantly with those showing alternative splicing using the hypergeometric overlap test available at http://nemates.org/MA/progs/overlap_stats.html. This analysis calculates a representation factor (RF), defined as the observed number of genes common to both lists divided by the expected number under random overlap. Of the 21,503 genes in the *V. maculifrons* genome, 16,673 with detectable expression were retained as the background universe for analysis. All genes with evidence of alternative splicing (> 1 splice variant) were also found to be differentially expressed. For overlap assessments, we defined three gene sets for both caste and tissue comparisons (Table S14): (1) differentially expressed genes (DEGs; *n* = 8,822 and 10,017, respectively); (2) genes with at least one alternative splicing (AS) event (*n* = 153 and 615, respectively); and (3) genes satisfying both criteria (*n* = 126 and 551, respectively). RF > 1 indicates enrichment beyond chance expectation, while RF < 1 indicates depletion. This approach addresses whether genes prone to expression changes are also prone to splicing changes.

Second, we evaluated whether the magnitude of transcriptomic responses, measured as the number of differentially expressed genes (DEGs) and the number of genes with alternative splicing events (AS genes), was coordinated across biological comparisons (Table S14). This analysis addresses a complementary question: when environmental or genetic factors produce large changes in gene expression across many genes, do they also produce large changes in alternative splicing? We calculated Spearman's rank correlation coefficient (*ρ*) between the number of DEGs and the number of AS genes across pairwise comparisons. Analyses were performed separately for colony‐level comparisons (*n* = 21 colony pairs) and patriline‐level comparisons (*n* = 9 patriline pairs within colonies) using R's cor.test(…, method = “spearman”). We calculated two‐tailed *p*‐values via Student's t‐distribution and, because we hypothesized a positive association a priori, also report one‐tailed *p*‐values (Table S15).

Together, these two approaches provide complementary perspectives on the relationship between gene expression and alternative splicing: the overlap analysis identifies whether the same genes show both types of regulatory changes, while the correlation analysis examines whether the overall magnitude of expression and splicing responses covary across biological contexts.

## 3 Results

### 3.1 Patterns of Gene Expression in Castes and Tissues

We investigated the factors affecting the transcriptome across multiple levels of biological organization in the social wasp, *V. maculifrons* (Figure 1). After quality control, our dataset included 251 individuals from seven colonies and 15 patrilines (2–3 patrilines per colony), balanced across social roles (118 queens, 133 workers) and anatomical regions (126 heads, 125 thoraces).

**FIGURE 2:**
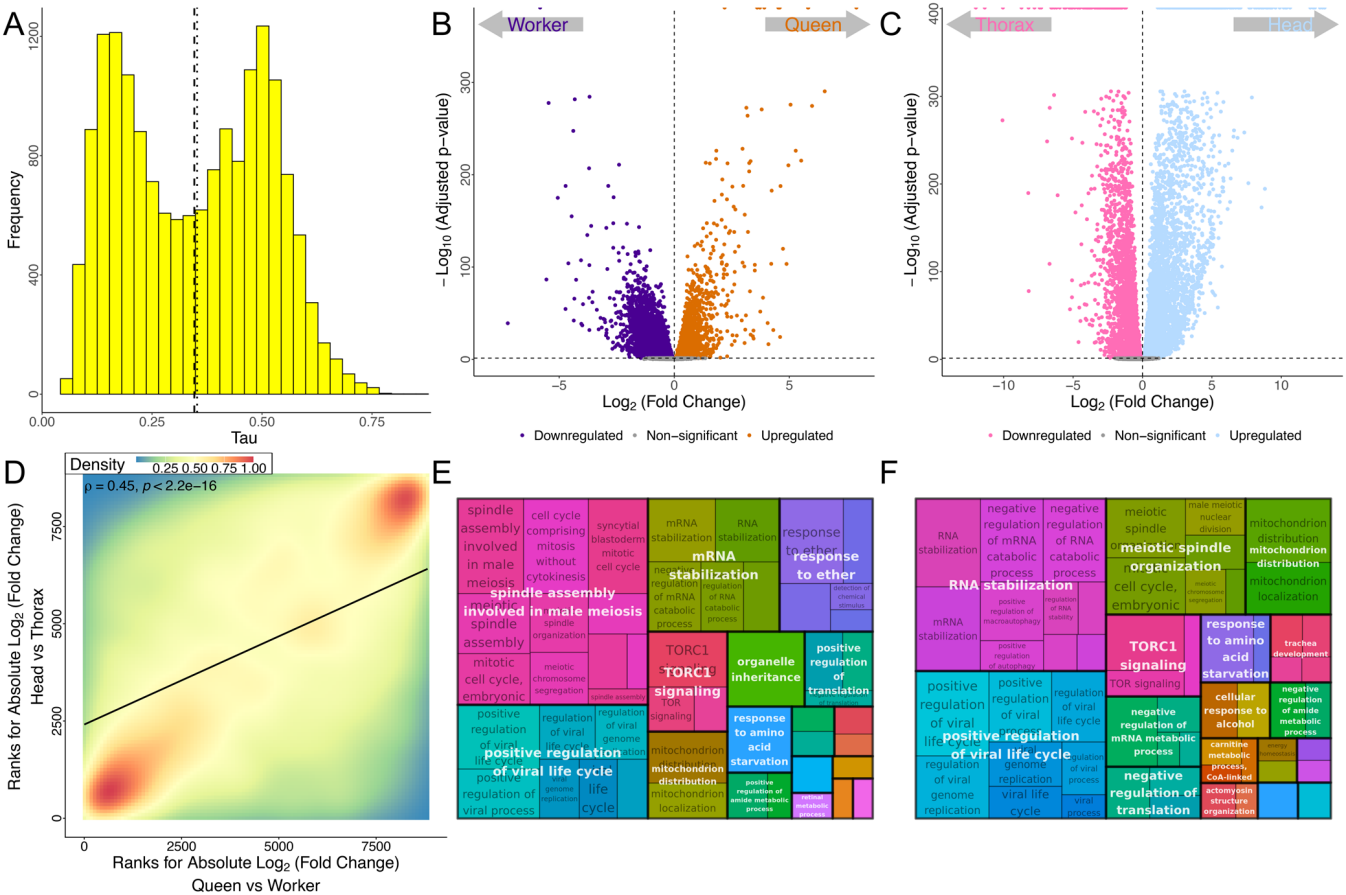
Analyses of differentially expressed genes. (A) Distribution of *τ* for all caste‐tissue combinations. The dashed (*τ* = 0.352) and dotted (*τ* = 0.346) lines represent the mean and median, respectively. (B) Differentially expressed genes between castes. (C) Differentially expressed genes between tissues. (D) Correlation between caste and tissue differential gene expression. The line shows the linear regression fit between log_2_ fold changes for caste (queen vs. worker) and tissue (head vs. thorax). (E) Gene ontology enrichment (Biological Processes) of differentially expressed genes between castes. (F) Gene ontology enrichment (Biological Processes) of differentially expressed genes between tissues.

To assess how broadly genes were expressed, we computed the *τ* specificity index for each gene across all samples (Yanai et al. 2005). We found that the resulting distribution of *τ* was bimodal (Figure 2A). The genes centered around the distribution displaying the lower mode of *τ* = 0.188 represented genes with relatively uniform expression across all samples and consisted of 8194 genes. The second mode peaks close to *τ* = 0.489 and consisted of 8479 genes showing more tissue‐specific expression.

We used a multivariable model that accounted for batch, patriline, caste, and tissue effects. Of the 16,673 genes with detectable expression, we identified 3247 genes significantly upregulated in queens and 5575 genes significantly upregulated in workers (Figure 2B). Caste‐biased genes were enriched for several biological processes (Figure 2E), reflecting the distinct physiological demands of each caste. Using the same multivariable model framework, differential expression between head and thoracic tissues revealed 5779 head‐biased and 4238 thorax‐biased genes (Figure 2C). Tissue‐biased genes were enriched for a variety of biological processes related to tissue function (Figure 2F). Our approach of using all genes that passed the false discovery rate (FDR) threshold allowed us to capture the full spectrum of regulatory changes that may be biologically relevant in social systems without filtering for Log_2_ Fold Change (LFC).

We were interested in determining if genes that were differentially expressed between castes tended to also be differentially expressed between tissues. We thus investigated the correlation between caste‐ and tissue‐biased expression. We found that the correlation between differential expression between castes and tissues was strongly and significantly positive (Figure 2D).

**FIGURE 3:**
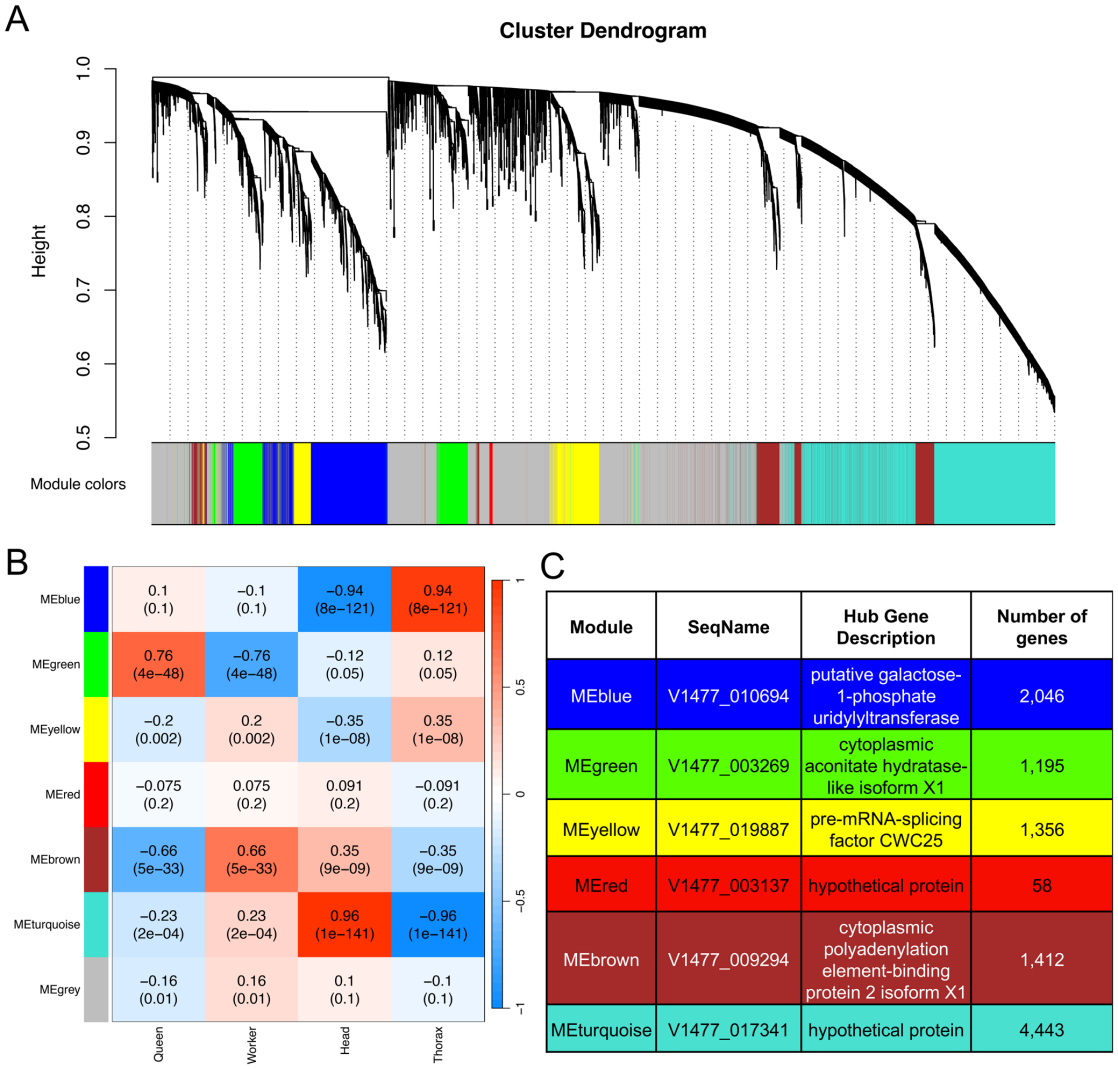
Co‐expression network analysis. (A) Dendrogram with module assignments showing which network each gene is clustered into. (B) Heatmap showing the correlation between module eigengenes and biological contexts. Categorical traits were encoded as binary numeric vectors (0/1) for Spearman correlation calculations. (C) Hub genes from each module and number of genes in each module.

We built a weighted gene co‐expression network to understand which genes were responsible for overall patterns of gene expression. Hierarchical clustering identified six modules (Figure 3A) ranging in size from 58 genes (yellow) to 4,443 genes (turquoise). Module eigengenes (the first principal component of each module) revealed associations between modules and biological variables; the green and brown modules were found to mostly relate to caste‐biased genes, while the turquoise and blue modules mostly related to tissue‐biased genes (Figure 3B). Hub genes, genes with the highest intramodular connectivity, showed variation in function across the modules (Figure 3C).

### 3.2 Alternative‐Splicing Landscape

**FIGURE 4:**
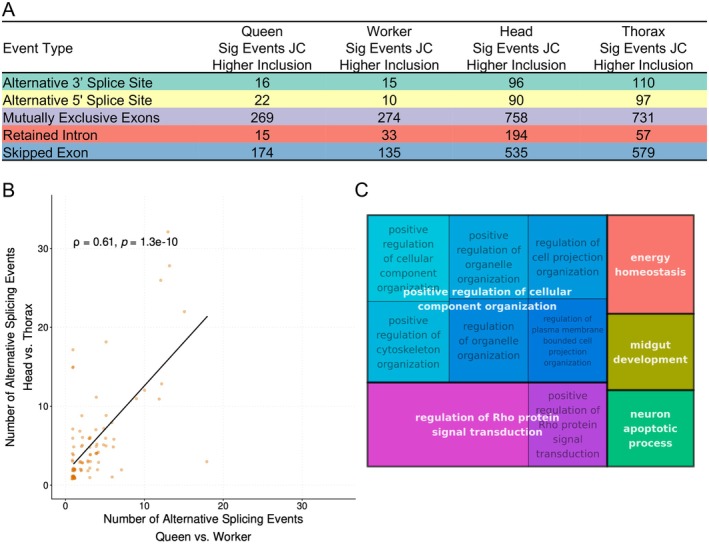
Alternative splicing events. (A) Comparison of the number of castes‐ and tissue‐biased alternative splicing events, defined as junction‐count (JC) events with significantly higher inclusion in the respective group. (B) Correlation between caste‐ and tissue‐biased number of alternative splicing events (*n* = 93). Spearman correlation and *p*‐value are indicated at the top of the figure. The solid black line represents the linear regression fit. Only genes with greater than zero splicing events in both contexts were considered. (C) Gene ontology enrichment under the category of biological processes of caste‐biased genes with alternative splicing events.

We investigated patterns of alternative splicing in *V. maculifrons* using pairwise group comparisons. We detected 963 significant splicing events between castes (Figure 4A). The types of splice events differed significantly between castes (*χ* ^2^ = 15.39, df = 4, *p* = 0.00396), indicating a non‐random distribution of alternative splicing event types. Queens were enriched for alternative 5′ splice sites (standardized residual = +1.36) and skipped exons (+1.18), whereas workers showed a strong excess of retained introns (+2.02).

We detected 3247 splicing events between head and thoracic tissues, substantially more than between castes (Figure 4A). The types of splice events also differed significantly between tissues, with divergence even more pronounced than between castes (*χ* ^2^ = 75.27, df = 4, *p* = 1.748e^−15^). Head tissue showed more retained introns than expected (standardized residual = +5.69), while thoracic tissue had relatively more skipped exons (+1.68). Other event types deviated only modestly from expectation (residuals ranging from ≈ −1.00 to +1.01).

We tested whether genes that were alternatively spliced between castes were also likely to be alternatively spliced between tissues. We found a strong, significant positive correlation between alternative splicing events across these contexts (Figure 4B), indicating that genes prone to splicing modulation in one biological context also tend to be alternatively spliced in another. Genes showing caste‐biased alternative splicing were significantly enriched for a set of biological processes (Figure 4C). However, tissue‐biased alternative splicing did not produce any significantly enriched GO categories.

### 3.3 Environmental and Genetic Effects on the Transcriptome

**FIGURE 5:**
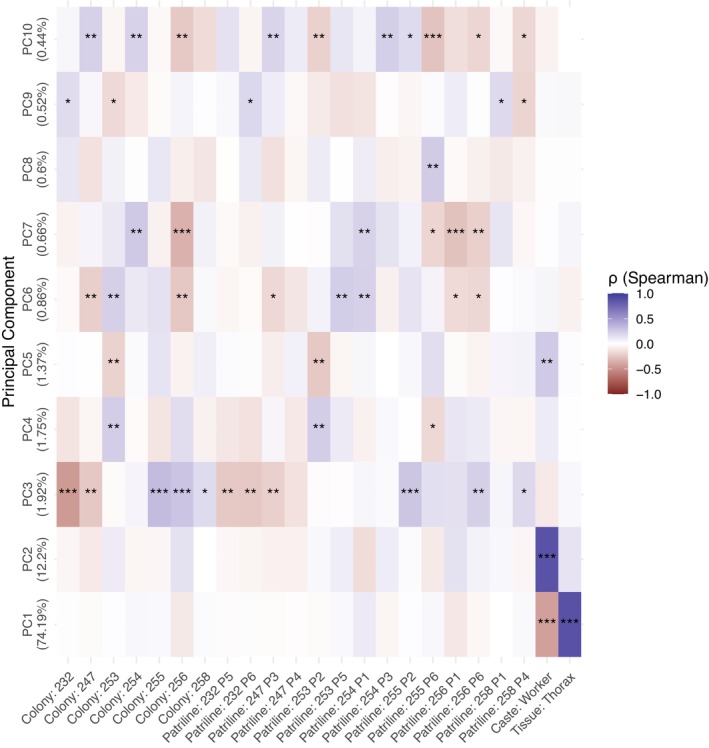
Principal components capture hierarchical sources of transcriptomic variation. Correlation between principal components (PC1–PC10) and sample attributes. Color intensity indicates correlation strength and direction (red = positive, blue = negative). PC1 and PC2 are dominated by tissue and caste effects respectively, while subsequent components reflect colony and patriline variation. Asterisks indicate statistical significance: * for *p* < 0.05, ** for *p* < 0.01, and *** for *p* < 0.001.

We investigated how genotype and environment influenced patterns of gene expression and alternative splicing. We first examined associations between principal components (PCs) and various study attributes (Figure 5). PC1 was strongly correlated with tissue, while PC2 was strongly correlated with caste. The remaining PCs were associated with various combinations of colony or patriline. Thus, overall, tissue and caste had the strongest effects on the patterns of gene expression. But colony and patriline also contributed to gene expression differences among samples.

**FIGURE 6:**
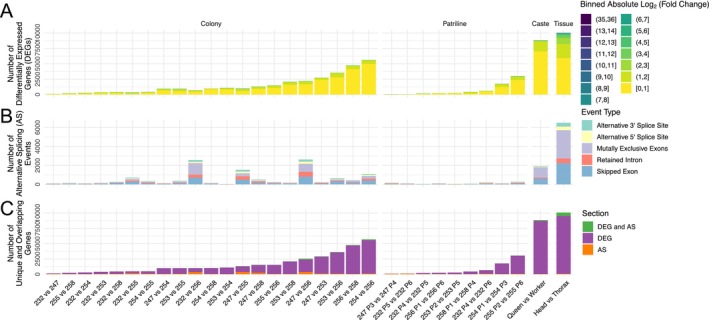
Comparisons of numbers of differentially expressed genes and number of alternative splicing events from various pairwise contexts arranged in ascending order by number of unique and overlapping genes. (A) Number of differentially expressed genes between groups. (B) Number of alternative splicing events between groups. (C) Number of unique and overlapping differentially expressed genes and alternatively spliced genes between groups.

We next determined the number of differentially expressed genes between individuals from different pairs of colonies and from different pairs of patrilines within colonies. In the colony‐wide dataset, differentially expressed gene numbers ranged from 100 to 5,736, whereas alternative splicing events varied between 23 and 485 (Figure 6A,B). Thus, there was substantial evidence for strong environmental effects on gene expression variation. Patriline‐specific comparisons exhibited a similarly broad differential gene expression profile (37–3000 genes) but consistently lower alternative splicing frequencies (18–73 events) (Figure 6A,B).

To assess whether sample size variation could explain the observed differences in transcriptomic responses, we examined correlations between sample size and the number of DEGs or AS events across comparisons. For colony‐level comparisons, sample size showed weak correlations with both DEG numbers (Spearman *ρ* = −0.404, *n* = 21) and AS gene numbers (Spearman *ρ* = 0.140), indicating that the substantial variation in transcriptomic responses (DEGs: 100–5,617; AS genes: 23–485) reflects genuine biological differences between colonies rather than sampling artifacts. Similarly, patriline comparisons showed weak correlations between sample size and DEG numbers (Spearman *ρ* = 0.749, *n* = 9) or AS events (Spearman *ρ* = 0.202), despite relatively consistent sample sizes (12–20 individuals per comparison).

We were interested in understanding the relationship between differential gene expression and alternative splicing (Figure 6C). We examined this question at two levels: first, whether the same genes show both types of regulatory changes, and second, whether the magnitude of expression and splicing responses are coordinated across biological comparisons. In the caste contrast, 1.4% of genes were both alternatively spliced and differentially expressed, with a representation factor of 1.6 (*p* = 1.725e^−14^), indicating almost twice as much overlap as expected by chance. In the tissue contrast, 5.5% of genes showed both types of changes, with a representation factor of 1.5 (*p* = 4.142e^−62^). These results indicate that genes prone to expression changes are also more likely to undergo splicing changes than would be expected if these regulatory mechanisms operated completely independently.

We next examined whether the overall magnitude of differential expression and alternative splicing responses were coordinated across multiple biological comparisons. At the colony level, we found a moderate positive correlation (Spearman *ρ* = 0.45, *p* = 0.042, *n* = 21 colony pairs), indicating that colony pairs showing many differentially expressed genes also tended to show many alternative splicing events. This suggests that environmental factors driving colony‐level transcriptomic divergence affect both expression levels and splicing patterns. In contrast, patriline comparisons within colonies showed a weak but non‐significant correlation (Spearman *ρ* = 0.26, *p* = 0.5, *n* = 9 patriline pairs), indicating that genetic differences between patrilines had more variable effects on these two regulatory mechanisms. Together, these findings reveal that differential expression and alternative splicing show partial concordance: the same genes are more likely than expected to show both types of changes.

## 4 Discussion

The phenotype of an organism is fundamentally shaped by its transcriptome. Differences in gene expression levels generate substantial intraspecific phenotypic variation, while alternative splicing expands the functional repertoire of each gene by producing multiple isoforms. Both processes are highly responsive to genetic background and environmental cues, providing complementary routes through which organisms respond to their surroundings.

In this study, we investigated gene expression and splicing variation in a highly social wasp to better understand factors that affect transcriptional variation that arises in the context of developmental plasticity. We specifically examined (i) the gene expression variation that distinguishes castes and tissues, (ii) the contribution of alternative splicing as an additional regulatory layer to tissue and caste differentiation, (iii) the interaction of gene expression and splicing variation, and (iv) the influence of genotype versus environment on the transcriptome.

### 4.1 Gene‐Expression Architecture Across Castes and Tissues

Queen and worker social insects share the same genome. However, they display markedly different life histories. Differences in gene expression between castes at least partially explain caste phenotypes, and recent studies have identified gene expression differences between castes (Feldmeyer et al. 2014; Ferreira et al. 2013; Grozinger et al. 2007; Korb et al. 2021; Orr and Goodisman 2023; Patalano et al. 2015; Qiu et al. 2022; Rittschof and Robinson 2016; Sumner 2014; Taylor et al. 2021; Taylor et al. 2019; Toga and Bono 2023; Vergara‐Martínez et al. 2025; Warner et al. 2019; Xu and Colgan 2025). Our expression profiling of *V. maculifrons* social wasp gene expression patterns revealed two broad categories of genes associated with caste phenotype. The first group comprises constitutively expressed genes with low tissue‐ and caste‐specificity that were expressed uniformly across all samples, forming a stable metabolic backbone that underpins basic cellular physiology (Joshi et al. 2022). These genes were expressed at similar levels across castes and tissues, consistent with core biological roles. The second group consisted of a more dynamic set of genes whose activity is tightly linked to a particular caste‐tissue combination.

Many genes show strong expression differences between castes. For example, caste‐biased genes were enriched for processes such as spindle and cytoskeletal organization, mRNA stabilization, nutrient signaling, and organelle dynamics. These enrichments reflect the distinct physiological contexts of the two castes (Kocher and Kingwell 2024). Prior studies in social insects have also documented caste differences in genes involved in pheromone transport, cuticular hydrocarbon biosynthesis, and neuropeptide activity (Berens et al. 2015; Feldmeyer et al. 2014; Orr and Goodisman 2023; Sumner et al. 2018).

We observed far more differentially expressed genes between tissues than between castes, which is expected given the profound functional specialization between the head and thoracic tissues (Li and White 2003; Mantica et al. 2024). Head tissue showed enrichment for genes related to RNA stabilization, regulation of mRNA processes, regulation of translation, and several stress and signaling responses, patterns that fit well with the high level of translational control and neural regulation required for sensory processing and social communication (Dickey et al. 2023; Fisher et al. 2025; Vieira et al. 2021; Whitfield et al. 2003; Zhao et al. 2016). In contrast, thoracic tissue exhibited elevated expression of genes involved in development, nutrient signaling, cytoskeletal organization, and cellular responses to stress, all of which are consistent with the high aerobic and mechanical demands of flight (Margotta et al. 2013; Soares et al. 2021). Similar expression patterns have been reported across insects, where tissue identity consistently explains more variance than developmental or social phenotypes (Xu and Colgan 2025). Together, these results indicate that caste identity modifies a deeply conserved tissue‐specific expression scaffold, fine‐tuning physiological programs to support the divergent roles of queens and workers.

Weighted gene co‐expression network analysis revealed that transcriptomic variation is organized into discrete functional modules, each associated with different biological factors (Figure 3). Caste‐associated modules (green and brown) contained genes showing coordinated expression differences between queens and workers, while tissue‐associated modules (turquoise and blue) comprised genes with coordinated tissue‐specific expression. Additional modules showed weaker associations with colony or patriline, indicating more subtle genetic and environmental effects. This modular organization suggests that phenotypic plasticity emerges from hierarchically structured gene networks, where core developmental programs (caste and tissue specification) involve tightly coordinated gene expression, while finer‐scale variation reflects more flexible, context‐dependent regulation. Such modularity could theoretically allow selection to act on specific regulatory networks without disrupting essential functions, though verifying this evolutionary trajectory will require comparative analyses beyond the scope of this study.

We investigated if genes that were differentially expressed between tissues also tended to be differentially expressed between castes. We found that there was indeed a very strong relationship between differential gene expression in these two contexts (Figure 2D). Thus, differential expression seems to be a property of genes themselves, such that some genes are able to show variation in expression across multiple contexts (Hunt et al. 2013; Warner et al. 2019; Witt et al. 2021; Xu and Colgan 2025). And the process of phenotypically plastic development appears to draw on the same types of genes in different circumstances (Buchberger et al. 2019; Hunt et al. 2011).

### 4.2 Alternative Splicing of Genes Across Castes and Tissues

While changes in overall gene expression dominate the transcriptional landscape, alternative splicing adds nuance by reshuffling exonic and intronic sequences to produce variant protein isoforms (Marasco and Kornblihtt 2023; Verta and Jacobs 2022; Wright et al. 2022). In our dataset, splicing events were considerably less numerous than differentially expressed genes. Nevertheless, there were a substantial number of genes that were alternatively spliced across castes or tissues.

We identified clear patterns of caste‐biased alternative splicing. Caste‐biased alternative splicing involved genes associated with positive regulation of cellular component organization, Rho protein signal transduction, energy homeostasis, midgut development, and neuronal apoptotic processes. These categories suggest that caste differences in isoform usage may influence cytoskeletal remodeling, intracellular signaling, and metabolic balance, which are all central to the divergent activity levels and physiological demands of queens and workers. The enrichment of pathways related to neuronal turnover and midgut function also hints that caste identity may shape how tissues maintain or remodel themselves across the colony cycle. These results suggest that caste differences extend beyond expression levels to include extensive isoform variation.

As was the case with patterns of differential gene expression, we found far more alternative splicing events between tissues than between castes. Tissue‐biased splicing was observed across many genes, although these events did not cluster into clearly enriched GO categories. The most striking pattern was the prevalence of retained introns in tissue‐specific comparisons, a phenomenon that echoes findings in other insects where intron retention fine‐tunes neuronal excitability (Grantham and Brisson 2018; The GTEx Consortium 2020). The transcriptome of workers was more heterogeneous compared to that of queens, as indicated by higher variance in gene expression across individuals.

We investigated whether genes that were alternatively spliced between castes were also alternatively spliced between tissues. We found that there was indeed a strong correlation between alternative splicing in these two contexts. Genes that are prone to splicing modulation in one context tend also to be spliced differently in another, hinting at an underlying regulatory architecture that predisposes certain loci to flexible post‐transcriptional control (Xu and Colgan 2025). This relationship between gene splicing in different contexts has not been investigated substantially and may represent an important consideration in our understanding of alternative splicing. The observed splicing results suggest a general phenomenon whereby genes that show flexibility in terms of splicing in one context also show flexibility in terms of another.

While we identified a significant overlap in genes undergoing alternative splicing across caste and tissue contexts, a comprehensive analysis of their structural and evolutionary features remains a priority for future comparative genomic studies. The current work establishes the existence and magnitude of this shared plasticity, providing a robust empirical foundation for such deeper investigations. These shared splicing candidates thus serve as a high‐confidence starting point for understanding the regulatory mechanisms that drive phenotypic diversity in social insects.

### 4.3 Relationship Between Gene Expression and Alternative Splicing

Our results demonstrated that genes that were differentially expressed between castes tended to also be differentially expressed between tissues. Similarly, genes that were alternatively spliced between castes tended to be alternatively spliced between tissues. This led us to explore if this pattern would extend across both expression level and splicing. That is, we investigated if a gene that was differentially expressed between biological contexts might also tend to be alternatively spliced in different contexts. If this were the case, then differential expression of genes and alternative splicing might be seen as complementary gene regulatory mechanisms. On the other hand, limited evidence for a correlation between differential gene expression and alternative splicing would support the view that these are contrasting regulatory systems.

We found evidence for coordination between differential expression and alternative splicing. Genes showing differential expression were significantly more likely to also be alternatively spliced than expected by chance, indicating that these regulatory mechanisms are not completely independent. However, most genes showed changes in only expression or splicing but not both, suggesting that while some genes utilize multiple regulatory layers for fine‐tuned control, expression and splicing predominantly operate on distinct gene sets. This regulatory architecture may provide flexibility for organisms to generate diverse phenotypic responses through complementary transcriptomic pathways.

Previous studies that have investigated the relationship between alternative splicing and differential expression in diverse taxa, including humans, salmonids, aphids, sticklebacks, and eusocial bees, have identified only limited concordance between the mechanisms. This suggests that expression changes often reflect broad shifts in metabolic or structural demands, whereas splicing refines functional protein variation without altering transcript abundance (Grantham and Brisson 2018; Jones et al. 2024; Luo et al. 2025; Marasco and Kornblihtt 2023; Menge and Sutherland 1987; Rodríguez‐Ramírez and Peichel 2025; Singh and Ahi 2022; The GTEx Consortium 2020; Verta and Jacobs 2022; Yamaguchi et al. 2022). Our findings therefore support a model in which expression and splicing represent partially concordant layers of gene regulation, each contributing in distinct ways to phenotypic plasticity (Verta and Jacobs 2022).

### 4.4 Genetic and Environmental Contributions to Transcriptomic Variation

A primary interest of this study was to gain a greater understanding of genetic and environmental effects on gene expression and alternative splicing variation. We took advantage of the polyandrous mating system of *V. maculifrons* to gain a greater understanding of genetic and environmental effects on the transcriptome. Individuals from different *V. maculifrons* colonies differ genetically and experience different environments. In contrast, individuals from different patrilines within colonies show genetic differences but are likely to experience a similar environment. Therefore, contrasting patterns of gene expression and alternative splicing between colonies and between patrilines within colonies provided some insight into how genotypic and environmental variation affected gene expression and splicing variation.

We found that individuals from different colonies exhibited surprisingly strong transcriptomic differences. Differences in gene expression between some colonies were extremely high. And remarkably, differences in alternative splicing between colonies exceeded the differences between castes in some cases (Figure 6A,B). The observed transcriptomic differences between colonies can arise from both genetic and environmental effects.

Environmental variation between colonies of *V. maculifrons* may take several forms. Colonies likely experience different microclimates, diets, ecological interactions, etc. Thus, our results demonstrate the powerful influence of environmental variation on transcriptomic variation (Cutter 2023; Hildebrandt et al. 2018; Logan and Cox 2020; Rivera et al. 2021; Schlichting and Pigliucci 1998).

Transcriptomic differences also arise, in part, from genetic differences between individuals from different colonies. *V. maculifrons* colonies are founded independently by single queens, each mated to multiple males. Thus, individuals from different colonies are unrelated and show substantial genetic differences. We found that genetic effects on the transcriptome, inferred by examining transcriptomic differences between patrilines within colonies, were relatively modest in most, albeit not all, colonies (Figure 6A,B). That is, gene expression and alternative splicing differences between patrilines within colonies varied widely but were small in most cases. In particular, there were very few splicing differences between patrilines, particularly relative to the differences between colonies. This indicates that genetic effects may not drive substantial splicing differences. While a complete separation of genetic and environmental effects would require a cross‐fostering experiment, our nested design allows us to robustly infer the dominant role of environmental variation by comparing the smaller genetic variance within colonies against the larger total variance between colonies.

Nevertheless, even when environmental variation in the transcriptome was minimized, a noticeable genotype‐driven component persisted, aligning with recent work in honeybees that shows colony genetics can set baseline expression levels for caste‐determining pathways (Barchuk et al. 2007; Bresnahan et al. 2025; Kocher and Kingwell 2024). Genetic effects on gene expression variation are widespread (Kang et al. 2023; Nica and Dermitzakis 2013; Signor and Nuzhdin 2018). Thus, both inherited genetic differences and the immediate ecological context jointly sculpt the transcriptional phenotype.

## 5 Conclusions

The purpose of this study was to understand the factors affecting transcriptomic variation associated with phenotypic plasticity in a social wasp. We found that tissue identity explained the largest portion of transcriptomic variation, with caste differences layered on top through more focused shifts in both expression levels and alternative transcript use. We also found that genes that were differentially expressed between social insect castes tended to be differentially expressed between tissues; notably, a similar correlation was observed for alternative splicing. This suggests that genes involved in developmental plasticity in one context are involved in other contexts as well. The relationship between differential gene expression and alternative splicing was more limited, albeit significant, indicating that these two mechanisms act somewhat concordantly in shaping phenotypic variation. We also found that colony‐level environmental variation contributed strongly to both regulatory processes, while genetic effects were present but generally weaker. The finding of strong environmental impacts on the transcriptome indicates that the environment can have very significant effects on phenotypic plasticity. By examining caste, tissue, colony, and patriline variation together, this study provides a more complete view of transcriptomic organization and highlights the value of integrating multiple regulatory layers to better understand phenotypic plasticity and social complexity.

## Author Contributions

**Michael A. Catto:** data curation (equal), formal analysis (equal), methodology (equal), software (equal), validation (equal), visualization (equal), writing – original draft (equal), writing – review and editing (equal). **Sarah E. Orr:** conceptualization (equal), data curation (equal), formal analysis (equal), investigation (equal), methodology (equal), resources (equal), writing – original draft (equal), writing – review and editing (equal). **Brendan G. Hunt:** project administration (equal), supervision (equal), writing – original draft (equal), writing – review and editing (equal). **Michael A. D. Goodisman:** conceptualization (equal), funding acquisition (equal), project administration (equal), supervision (equal), writing – original draft (equal), writing – review and editing (equal).

## Funding

This research was supported by the United States Department of Agriculture Agriculture and Food Research Initiative Education and Workforce Development Program, project award no. 2023‐67012‐39886 to SEO; and the United States National Science Foundation award no. 2105033 to MADG.

## Conflicts of Interest

The authors declare no conflicts of interest.

## Supporting information


**Table S1:** PCR primers used in this study.
**Table S2:** Microsatellites per sample.
**Table S3:** Sample information detailing the per sample ID, group heirarchy, and additional information.
**Table S4:** Raw read stats per each samples' paired end reads.
**Table S5:** Trimmed read stats per each samples' forward and reverse paired and unpaired reads.
**Table S7:** Mapping rates for each samples' trimmed paired end reads mapped to the *V. maculifrons* genome assembly.
**Table S6:** Principal component analysis data of all samples used for downstream analyses and all samples including 247p3W3T.
**Table S8:** Gene Tau calculations.
**Table S9:** DESeq2 by colony (~Batch + Colony + Caste + Tissue).
**Table S10:** DESeq2 by patriline (~Batch + Patriline + Caste + Tissue).
**Table S11:** WGCNA genes, modules, and connectivity values.
**Table S12:** rMATS number of alternative splicing events per gene.
**Table S13:** GO terms enrichment profiles.
**Table S14:** Group sample frequencies and overlap numbers.
**Table S15:** DEG and AS overlap hypermatrix correlations.

## Data Availability

The datasets generated and analyzed during the current study have been deposited to the National Center for Biotechnology Information (NCBI) and Dryad. The NCBI data can be found under the BioProject PRJNA1122603, which includes SRA accessions SRR29419127–SRR29419376 and BioSample accessions SAMN41802038–SAMN41802288. Scripts and additional data can be found in the Dryad repository DOI: 10.5061/dryad.g1jwstr5k and in the Zenodo repository DOI: 10.5281/zenodo.21075120. Tables S1–S15 can be found in the Supporting Information file.
